# Scalable Production of Human Mesenchymal Stromal Cell-Derived Extracellular Vesicles Under Serum-/Xeno-Free Conditions in a Microcarrier-Based Bioreactor Culture System

**DOI:** 10.3389/fcell.2020.553444

**Published:** 2020-11-03

**Authors:** Miguel de Almeida Fuzeta, Nuno Bernardes, Filipa D. Oliveira, Ana Catarina Costa, Ana Fernandes-Platzgummer, José Paulo Farinha, Carlos A. V. Rodrigues, Sunghoon Jung, Rong-Jeng Tseng, William Milligan, Brian Lee, Miguel A. R. B. Castanho, Diana Gaspar, Joaquim M. S. Cabral, Cláudia Lobato da Silva

**Affiliations:** ^1^iBB-Institute for Bioengineering and Biosciences and Department of Bioengineering, Instituto Superior Técnico, Universidade de Lisboa, Lisbon, Portugal; ^2^Instituto de Medicina Molecular, Faculdade de Medicina, Universidade de Lisboa, Lisbon, Portugal; ^3^Centro de Química Estrutural and Department of Chemical Engineering, Instituto Superior Técnico, Universidade de Lisboa, Lisbon, Portugal; ^4^PBS Biotech Inc., Camarillo, CA, United States; ^5^AventaCell Biomedical Corp., Atlanta, GA, United States

**Keywords:** extracellular vesicles, mesenchymal stromal cells (MSC), scalable production, bioreactors, serum-/xenogeneic-free

## Abstract

Mesenchymal stromal cells (MSC) hold great promise for tissue engineering and cell-based therapies due to their multilineage differentiation potential and intrinsic immunomodulatory and trophic activities. Over the past years, increasing evidence has proposed extracellular vesicles (EVs) as mediators of many of the MSC-associated therapeutic features. EVs have emerged as mediators of intercellular communication, being associated with multiple physiological processes, but also in the pathogenesis of several diseases. EVs are derived from cell membranes, allowing high biocompatibility to target cells, while their small size makes them ideal candidates to cross biological barriers. Despite the promising potential of EVs for therapeutic applications, robust manufacturing processes that would increase the consistency and scalability of EV production are still lacking. In this work, EVs were produced by MSC isolated from different human tissue sources [bone marrow (BM), adipose tissue (AT), and umbilical cord matrix (UCM)]. A serum-/xeno-free microcarrier-based culture system was implemented in a Vertical-Wheel^TM^ bioreactor (VWBR), employing a human platelet lysate culture supplement (UltraGRO^TM^-PURE), toward the scalable production of MSC-derived EVs (MSC-EVs). The morphology and structure of the manufactured EVs were assessed by atomic force microscopy, while EV protein markers were successfully identified in EVs by Western blot, and EV surface charge was maintained relatively constant (between −15.5 ± 1.6 mV and −19.4 ± 1.4 mV), as determined by zeta potential measurements. When compared to traditional culture systems under static conditions (T-flasks), the VWBR system allowed the production of EVs at higher concentration (i.e., EV concentration in the conditioned medium) (5.7-fold increase overall) and productivity (i.e., amount of EVs generated per cell) (3-fold increase overall). BM, AT and UCM MSC cultured in the VWBR system yielded an average of 2.8 ± 0.1 × 10^11^, 3.1 ± 1.3 × 10^11^, and 4.1 ± 1.7 × 10^11^ EV particles (*n* = 3), respectively, in a 60 mL final volume. This bioreactor system also allowed to obtain a more robust MSC-EV production, regarding their purity, compared to static culture. Overall, we demonstrate that this scalable culture system can robustly manufacture EVs from MSC derived from different tissue sources, toward the development of novel therapeutic products.

## Introduction

Mesenchymal stromal cells (MSC) exhibit multilineage differentiation ability, as well as intrinsic immunomodulatory and trophic activities, standing as promising candidates for tissue engineering and cell-based therapies (Caplan and Dennis, 2006; da Silva Meirelles et al., 2009). MSC are able to inhibit apoptosis and scarring (fibrosis), promote angiogenesis and support growth and differentiation of progenitor cells into functional regenerative units (Caplan and Dennis, 2006; da Silva Meirelles et al., 2009). The array of beneficial effects attributed to MSC has made them one of the most studied cells in clinical trials (Heathman et al., 2015). The trophic activity of MSC relies greatly on the secretion of bioactive factors that assist in repair and regeneration processes through paracrine signaling (Caplan and Dennis, 2006; da Silva Meirelles et al., 2009).

Recently, increasing evidence suggests that several MSC-associated paracrine therapeutic features are mediated by extracellular vesicles (EVs) (Bruno et al., 2009; Lai et al., 2010; Lener et al., 2015; Börger et al., 2017). EVs, such as exosomes and microvesicles, are lipid membrane enclosed structures actively secreted by cells. These vesicles have emerged as relevant mediators of intercellular communication, through the transfer of a cargo of proteins and RNA (i.e., microRNA and mRNA), which trigger alterations on host cells (Raposo et al., 1996; Ratajczak et al., 2006; Valadi et al., 2007). Their small size (generally 50 – 1000 nm) and resemblance to the cell membrane makes EVs ideal candidates to cross biological barriers, thus providing high biocompatibility to target cells (Alvarez-Erviti et al., 2011; El Andaloussi et al., 2013; Van Niel et al., 2018).

EV can be used in therapeutic settings through two different approaches. On one hand, EVs are able to mediate some of the therapeutic effects from their cells of origin (Lai et al., 2010; Bruno et al., 2012). Therefore, EVs could be potentially used in substitution of their cell of origin, as a cell-free therapy triggering equivalent therapeutic effect. On the other hand, EVs can be used as drug delivery vehicles, by loading EVs with therapeutic cargo, as an alternative to synthetic drug delivery systems (Batrakova and Kim, 2015).

Mesenchymal stromal cells are particularly interesting for EV production for a number of reasons. MSC are considered immune evasive cells and the safety of their administration has already been confirmed in a number of clinical trials (Lalu et al., 2012). Therefore, it is reasonable to assume that MSC-derived EVs (MSC-EVs) are not prone to immune reaction from the host immune system (Mendt et al., 2018; Elahi et al., 2020), and promising for the development of allogeneic (i.e., off-the-shelf) therapeutic products. MSC are intrinsically therapeutic, with promising applications for multiple diseases and MSC-EVs convey similar benefits as well (Lener et al., 2015; Phinney and Pittenger, 2017). Finally, MSC show great ability for expansion when cultured *ex vivo* and robust expansion platforms have already been established (Rafiq et al., 2013; dos Santos et al., 2014; Schirmaier et al., 2014; Carmelo et al., 2015; Mizukami et al., 2016; Lawson et al., 2017).

Despite the promising potential of EVs for therapeutic applications, robust manufacturing processes that would increase the consistency and scalability of EV production are still lacking. Similarly to the cell therapy context, where large cell numbers per dose are required (Ren et al., 2012; Golpanian et al., 2016; Wysoczynski et al., 2018), very large numbers of EVs are expected to be required for clinical use (e.g., each patient may require 0.5 – 1.4 × 10^11^ EVs, Kordelas et al., 2014). In order to achieve such large production capacities, robust and scalable manufacturing processes need to be developed.

The development of cell-based therapies faces multiple challenges (recently reviewed de Almeida Fuzeta et al., 2019) and these also apply to manufacturing of EV products. One of these challenges is the use of appropriate cell culture medium. The most commonly used culture medium supplement in *ex vivo* expansion platforms of MSC is fetal bovine serum (FBS), which presents several disadvantages when considering the production of cell-based therapies for human use due to their animal origin. As an alternative to animal derived products, serum-/xenogeneic-free (S/XF) culture supplements have been developed, such as human platelet lysates (hPL).

Another major challenge is determining the appropriate cell culture platform for scalable manufacturing of cell-based therapies (de Almeida Fuzeta et al., 2019). In order to achieve large product batches for clinical use, culture platforms require scalability as well as the ability to monitor and control culture parameters, which cannot be accomplished in traditional static culture systems. Multiple bioreactor configurations operating in dynamic culture conditions have been developed for this purpose (de Soure et al., 2016; de Almeida Fuzeta et al., 2019). Expansion of MSC immobilized on microcarriers has been explored in stirred-tank bioreactor configurations (de Soure et al., 2016; de Almeida Fuzeta et al., 2019). These bioreactors use an agitation system to maintain microcarriers in suspension and allow medium homogenization. However, agitation impacts cellular physiology due to increased shear stress.

In order to improve agitation patterns in cell culture, PBS Biotech has developed scalable Vertical-Wheel^TM^ bioreactors (VWBR) that can provide gentle and uniform mixing with minimal shear stress. A vertically rotating wheel promotes radial and axial fluid flow and creates a more homogeneous hydrodynamic environment compared with traditional stirred-tank bioreactors. In addition, the Vertical-Wheel^TM^ impeller can fully suspend microcarriers with minimal power input and thus minimize shear stress effects (Croughan et al., 2016). Moreover, this technology is scalable, being available at working volumes that range from 100 mL up to 500 L. Recently, VWBR have been successfully applied in microcarrier-based cell culture processes for the expansion of MSC from multiple sources (Sousa et al., 2015; de Sousa Pinto et al., 2019), as well as for human induced pluripotent stem cells (Rodrigues et al., 2018; Nogueira et al., 2019).

In this work, EVs were produced by MSC isolated from different human tissue sources, namely bone marrow (BM), adipose tissue (AT), and umbilical cord matrix (UCM). A S/XF microcarrier-based culture system was implemented in a single-use VWBR, employing a hPL culture supplement (UltraGRO^TM^-PURE), toward the production of MSC-EVs.

When compared with traditional static culture systems (i.e., T-flasks), the bioreactor-based culture system allowed a substantial improvement in EV production. This culture system is expected to contribute to robustly manufacture human MSC-EVs in a scalable manner, which can be applied as intrinsic medicines or as delivery vehicles in different therapeutic settings.

## Materials and Methods

### MSC Isolation From Human Samples

Human MSC used in this study are part of the cell bank available at the Stem Cell Engineering Research Group (SCERG), iBB-Institute for Bioengineering and Biosciences at Instituto Superior Técnico (IST). MSC were previously isolated/expanded according to protocols previously established at iBB-IST. UCM MSC were isolated in hPL-supplemented medium according to the protocol described by de Soure et al. (2017). BM MSC were isolated in hPL-supplemented medium by adapting the protocol for cell isolation using FBS-supplemented medium described by dos Santos et al. (2010). AT MSC were originally isolated in FBS-supplemented medium according to Oliveira et al. (2012), cryopreserved and later adapted for 1 or 2 passages to hPL-supplemented medium. Originally, human tissue samples were obtained from local hospitals under collaboration agreements with iBB-IST (bone marrow: Instituto Português de Oncologia Francisco Gentil, Lisboa; adipose tissue: Clínica de Todos-os-Santos, Lisboa; umbilical cord: Hospital São Francisco Xavier, Lisboa, Centro Hospitalar Lisboa Ocidental, Lisboa). All human samples were obtained from healthy donors after written informed consent according to the Directive 2004/23/EC of the European Parliament and of the Council of 31 March 2004 on setting standards of quality and safety for the donation, procurement, testing, processing, preservation, storage and distribution of human tissues and cells (Portuguese Law 22/2007, June 29), with the approval of the Ethics Committee of the respective clinical institution. Human MSC from the different sources (BM, AT, and UCM) were cryopreserved in a liquid/vapor-phase nitrogen container.

### MSC Expansion in Static Conditions

In general, MSC expansion in static conditions was performed as previously described (de Sousa Pinto et al., 2019). In summary, previously isolated BM, AT and UCM MSC were thawed and plated on T-flasks (Falcon), at a cell density between 3000–6000 cell/cm^2^. MSC were cultured in low glucose (1 g/L) Dulbecco’s Modified Eagle’s Medium (DMEM) (Gibco, Life Technologies), supplemented with 5% v/v of the human platelet lysate (hPL) UltraGRO^TM^-PURE (AventaCell Biomedical) and Antibiotic-Antimycotic (1x) (Gibco, Life Technologies).

Cells were maintained at 37°C and 5% CO_2_ in a humidified atmosphere and culture medium was changed every 3–4 days. At 70–80% cell confluence, MSC were detached from the flasks using the xeno-free cell detachment solution TrypLE^TM^ Select (1x) (Gibco, Life Technologies) for 7 min at 37°C. Cell number and viability were determined using the Trypan Blue (Gibco, Life Technologies) exclusion method.

**FIGURE 1:**
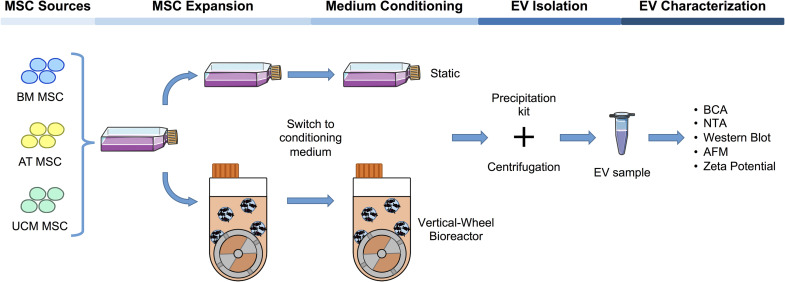
Workflow of the production and characterization of MSC-EVs in bioreactors and static systems. MSC were isolated from three different human tissue sources: BM, AT, and UCM. Firstly, MSC were expanded in static conditions (i.e., T-flasks) in hPL supplemented DMEM. These cells were subsequently used to inoculate a VWBR (5M cells; 100 mL final working volume), as well as to maintain a static culture in T-175 flasks. For each cell source, MSC from three independent donors (*n* = 3; BM1, 2, 3; AT1, 2, 3; UCM1, 2, 3) were used to inoculate either the final T-flasks for EV production or the VWBR, in passages from P4 to P5 [specifically, BM1 (P4); BM2 (P5); BM3 (P4); AT1 (P4); AT2 (P4); AT3 (P5); UCM1 (P4); UCM2 (P4); UCM3 (P5)]. Upon reaching stationary growth phase in VWBR or maximum confluency in static, the culture medium was changed for supplement-free culture medium and culture was maintained for 48 h. Over this period, culture medium was enriched in EVs secreted by cultured MSC. This conditioned culture medium was recovered and EVs were isolated by precipitation using a commercially available kit. Finally, EV production was quantified in both static and dynamic systems and samples were characterized using multiple techniques. MSC, mesenchymal stromal cells; EV, extracellular vesicles; VWBR, Vertical-Wheel^TM^ bioreactor; hPL, human platelet lysate; BM, bone marrow; AT, adipose tissue; UCM, umbilical cord matrix; NTA, nanoparticle tracking analysis; AFM, atomic force microscopy. The cells, T-flask and Eppendorf cartoons were obtained from Smart Servier Medical Art (https://smart.servier.com).

After thawing, MSC were passaged at least once before either final inoculation in T-flasks for EV production under static conditions or inoculation in VWBR. MSC were always plated at 3000 cell/cm^2^. For each cell source, MSC from three independent donors (*n* = 3) in passages (P) from P4 to P5 were used to inoculate either the final T-flasks for EV production or the VWBR [specifically, BM1 (P4); BM2 (P5); BM3 (P4); AT1 (P4); AT2 (P4); AT3 (P5); UCM1 (P4); UCM2 (P4); UCM3 (P5)] (Figure 1).

### MSC-EV Production Under Static Conditions

For the production of MSC-EVs under static conditions, previously cultured MSC were passaged to T-175 flasks, at 3000 cells/cm^2^. Cells were cultured in the same conditions described before for MSC expansion under static conditions. When maximum cell confluency in the flasks was achieved (90–100%), cells were washed once with basal DMEM low glucose (i.e., supplemented only with Antibiotic-Antimycotic) and subsequently cultured for 48 h in basal DMEM low glucose (20 mL per T-175), for medium conditioning. At the end of the 48 h period, the conditioned medium was recovered, centrifuged (360 × *g*, 10 min) to remove cell debris and stored at 4°C for less than 1 week until processing for EV isolation.

After recovery of the conditioned medium, MSC were detached from the flasks and cell number was determined as previously described. Cells were re-suspended in phosphate-buffered saline (PBS) for pelleting and stored at −80°C until further analysis (i.e., Western blots).

### MSC Expansion and MSC-EV Production in the Bioreactor Culture System

Expansion of human MSC in VWBR was generally performed as previously described (de Sousa Pinto et al., 2019). In summary, previously isolated and expanded human MSC were inoculated in a PBS 0.1 MAG bioreactor (PBS Biotech Inc.) with a working volume of 100 mL. Animal product-free SoloHill plastic microcarriers (PALL) were used in order to provide a surface for MSC to adhere and proliferate. Inoculation in the VWBR was performed in 60 mL of the same culture medium used for static conditions (i.e., DMEM low glucose, 5% v/v UltraGRO^TM^-PURE, Antibiotic-Antimycotic 1x), with an initial MSC number of 5 × 10^6^ and 2 g of microcarriers. The VWBR was placed at 37°C and 5% CO_2_ in a humidified atmosphere.

After an initial intermittent agitation regime, a continuous agitation mode was set at 25 rpm, as previously described (de Sousa Pinto et al., 2019). This agitation rate was always maintained, except for AT MSC culture, which required an increment in the agitation rate to 30 rpm at day 2 or 3 of culture and to 35 rpm at day 4 or 5, due to increased medium viscosity and the subsequent formation of cell aggregates.

After 2 days of culture, 40 mL of fresh culture medium with a glucose pulse (3 g/L) was added to the VWBR, achieving a final working volume of 100 mL. From this day onward, 25% v/v of culture medium was exchanged every 24 h, with the addition of fresh culture medium supplemented with a glucose pulse (3 g/L). Cell growth and viability were assessed every day, as previously described (de Soure et al., 2017). Growth rate was determined by performing an exponential fitting to experimental data corresponding to the exponential growth phase. Cell visualization on microcarriers was performed by staining the cells with 4′,6-diamidino-2- phenylindole (DAPI, Sigma, 1.5 μg/mL in PBS), as previously described (de Soure et al., 2017).

When MSC cultures reached stationary growth and the maximum cell concentration was achieved, the MSC expansion stage of the process was concluded and the EV production stage started. The culture medium was removed from the VWBR, after a 10 min sedimentation of cells attached to microcarriers inside the vessel. The VWBR was washed with 60 mL basal DMEM low glucose medium, at 30 rpm agitation, in order to remove hPL components. The cells on microcarriers were sedimented once again for 10 min and the washing medium was removed. MSC were kept in culture in the VWBR for 48 h in 60 mL basal DMEM low glucose medium, in the same conditions (i.e., agitation speed, temperature, O_2_ and CO_2_ concentrations) used for MSC expansion.

At the end of the 48 h period, the whole culture volume was recovered from the VWBR and transferred to 50 mL tubes (Falcon), where cells on microcarriers were sedimented for 10 min. The MSC conditioned medium was recovered and centrifuged at 360 × *g* for 10 min, to remove remaining microcarriers, cells and cell debris. Conditioned medium was stored at 4°C for less than 1 week until processing for EV isolation. After recovery of the conditioned medium, cells attached to microcarriers were re-suspended in PBS and stored at −80°C for further analysis (i.e., Western blots).

### Isolation of EVs From MSC Cultures

Extracellular vesicles were isolated using the Total Exosome Isolation reagent (Invitrogen, Life Technologies), according to the manufacturer instructions. Briefly, MSC conditioned medium was centrifuged for 30 min at 2000 × *g*, to remove cell debris and incubated overnight at 4°C with the isolation reagent. This mixture was then centrifuged for 1 h at 10000 × *g* and 4°C. The supernatant was discarded and the EV fraction was recovered by thoroughly washing the walls of the centrifuge tube with PBS 1x (Invitrogen, Life Technologies) in UltraPure^TM^ DNase/RNase-Free Distilled Water (Invitrogen, Life Technologies). EV samples were re-suspended in a PBS volume corresponding to a concentration factor of 20x to 70x relatively to the processed conditioned medium volume. EV samples were frozen at −80°C in aliquots (50–100 μL), in order to minimize freeze-thawing cycles.

### Comprehensive Characterization of Manufactured EVs

#### Protein Quantification

Total protein was quantified in EV samples using the Pierce^TM^ BCA Protein Assay Kit (Thermo Scientific), according to manufacturer instructions for the microplate procedure. Samples were quantified either undiluted or after a 2x dilution. Three replicates were quantified for each sample. Sample concentration was determined by applying a linear fit to the bovine serum albumin (BSA) standards and using the resulting equation to determine each sample concentration from its absorbance measurement.

#### Nanoparticle Tracking Analysis

EV size distribution profiles and concentration measurements were obtained by nanoparticle tracking analysis (NTA), using a NanoSight LM14c instrument equipped with a 405 nm laser (Malvern) and NTA software version 3.1 (Malvern). Silica 100 nm microspheres (Polysciences, Inc.) were routinely analyzed to check instrument performance (Gardiner et al., 2013). NTA acquisition and post-acquisition settings were optimized and kept constant for all samples. These settings were established using silica 100 nm microspheres (Gardiner et al., 2013) and subsequently adjusted for optimal detection of MSC-EVs.

EV samples were diluted in 2 mL of PBS 1x in UltraPure^TM^ DNase/RNase-Free Distilled Water, to obtain a final concentration in the range of 5 × 10^8^ to 3 × 10^9^ particles/mL. Samples were measured using a camera level of 13. Acquisition temperature was controlled and maintained at 20°C. Each sample was recorded 10 times for 30 s, using fresh sample for each acquisition (by pushing the sample syringe). The detection chamber was thoroughly washed with PBS between each sample measurement. A threshold level of 7 was applied for video processing. Each video recording was analyzed to obtain the size and concentration of EVs.

#### Western Blot

Cells were lysed with Catenin lysis buffer (1% Triton X-100, Sigma, 1% Nonidet P-40, Sigma, in PBS) supplemented with protease inhibitor (Sigma) and phosphatase inhibitor (Sigma) for 10 min on ice and then centrifuged at 14000 × *g* for 10 min at 4°C to remove insoluble material. Supernatants were recovered and used as whole cell lysates (WCL). For CD63 and CD81 detection, cells and EV samples were lysed with RIPA lysis buffer (150 mM NaCl, 25 mM Tris pH 7.4, 1% Nonidet P-40, 0.5% sodium deoxycholate, 0.1% SDS) and sonicated (three rounds of 5 s, at 50% intensity). Total protein content in WCL and EV samples was quantified using the BCA kit as previously described.

Both WCL and EV samples were mixed with sample buffer in reducing conditions and heated to 100°C for 10 min. For CD63 and CD81 detection, urea containing sample buffer was used. All samples were loaded (6–30 μg of total protein) in 4–12% Bis–Tris polyacrylamide gels (Invitrogen, Life Technologies), in equal protein content for each gel, and subjected to electrophoresis.

Proteins were transferred into nitrocellulose membranes using a Power Blotter System (Invitrogen, Life Technologies). Membranes were blocked with 5% w/v non-fat dry milk solution in tris-buffered saline (TBS) Tween 20 buffer 1x (Thermo Fisher Scientific), for 1 h with mild orbital agitation at room temperature and incubated with primary antibodies overnight at 4°C. For CD63 and CD81 detection, membranes were blocked with 5% BSA solution in TBS Tween 20 buffer 1x. Finally, membranes were incubated with HRP conjugated secondary antibodies for 1 h at room temperature and Pierce^TM^ ECL Western Blotting Substrate (Thermo Fisher Scientific) was applied for membrane revelation.

Primary antibodies included anti-Calnexin (1:1000, BD), anti-Synthenin (1:1000, Abcam), anti-CD63 (1:1000, Genetex), anti-CD81 (1:500, Abcam) and anti-GAPDH (1:1000, Santa Cruz). Secondary antibodies included Goat anti-Mouse IgG (H + L) Cross-Adsorbed Secondary Antibody, HRP (1:5000, Invitrogen, Life Technologies) and Goat anti-Rabbit IgG HRP-conjugated (1:1000, R&D Systems). Image acquisition was performed on iBright^TM^ CL1500 Imaging System (Invitrogen, Life Technologies).

#### Atomic Force Microscopy Imaging

EV samples were prepared for atomic force microscopy (AFM) imaging in freshly cleaved mica without any previous dilution. A volume ranging between 30–70 μL was used and samples were allowed to deposit during 30 min to 2 h. After this period, the samples were washed with filtered MilliQ water and air dried. AFM imaging was performed with a JPK Nano Wizard IV mounted on a Zeiss Axiovert 200 inverted microscope (Carl Zeiss). The AFM head is equipped with a 15 μm z-range linearized piezoelectric scanner and an infrared laser. Uncoated silicon ACL cantilevers from AppNano were used, with resonance frequencies and spring constants ranging between 160–225 kHz and 36–90 N/m, respectively. Scan speeds were between 0.1 and 0.3 Hz. Total areas with 10 × 10 μm were scanned with a 512 × 512 pixel resolution, in AC mode. Height and error images were recorded, and line fitted. Image processing was performed on JPK SPM data processing software version spm-6.0.55.

#### Zeta Potential

EV samples were diluted to a final protein concentration of 25 μg/mL, in PBS. Samples were loaded into disposable zeta cells with gold electrodes and allowed to equilibrate for 15 min at 37°C. Zeta potential measurements consisted in a set of 15 runs, each one resulting from an automatically defined number of subruns (ranging from 10 to 100) performed on the Zetasizer Nano ZS (Malvern), at a constant voltage of 40 V.

### Lactate Dehydrogenase Activity Measurements

Cell culture medium samples from VWBR cultures were recovered daily and centrifuged at 360 × *g* for 10 min, to remove remaining microcarriers, cells and cell debris. Lactate dehydrogenase (LDH) activity was quantified in cell culture supernatants using the Pierce LDH Cytotoxicity Assay Kit (Thermo Scientific) by adapting the manufacturer instructions for the microplate procedure. The same procedure was applied to a positive control (1 μL LDH Positive Control in 10 mL of 10% BSA in PBS). Three replicates were quantified for each sample. The LDH activity was reported as the quotient between the LDH activity of each sample and the LDH activity of the positive control, according with the following equation.

$$LDHactivity\left(\%\right)=\frac{L\,D\,H_{s\,a\,m\,p\,l\,e}}{L\,D\,H_{p\,o\,s.c\,o\,n\,t\,r\,o\,l}}\times 100$$

### Statistical Analysis

Statistical analysis was performed using GraphPad Prism 8 Software. Results are presented as mean ± standard error of the mean (SEM) of the values obtained from different MSC donors (i.e., biological replicates) or as mean ± standard deviation (SD) of the values from technical replicates. Paired *t*-test was applied to evaluate the statistical significance of the differences in EV concentration and specific EV concentration in the conditioned medium from MSC cultures in static and VWBR systems. These data sets passed normality tests. *P*-values result from two-tailed tests with a 95% confidence interval. Differences were considered significant at *P* < 0.05 and statistical output was represented as ^∗∗^ < 0.01.

## Results

### MSC Expansion and Medium Conditioning for MSC-EV Production From Three Different Human Sources (BM, AT and UCM) Was Achieved in the Bioreactor Culture System

Bioreactors have been implemented as scalable platforms for MSC manufacturing. Building on previous work from our group (de Sousa Pinto et al., 2019), a S/XF microcarrier-based culture system implemented in a VWBR originally targeting MSC expansion was adapted to the production of cell-derived products such as MSC-EVs and compared with traditional static culture systems (i.e., T-flasks) (Figure 1).

**FIGURE 2:**
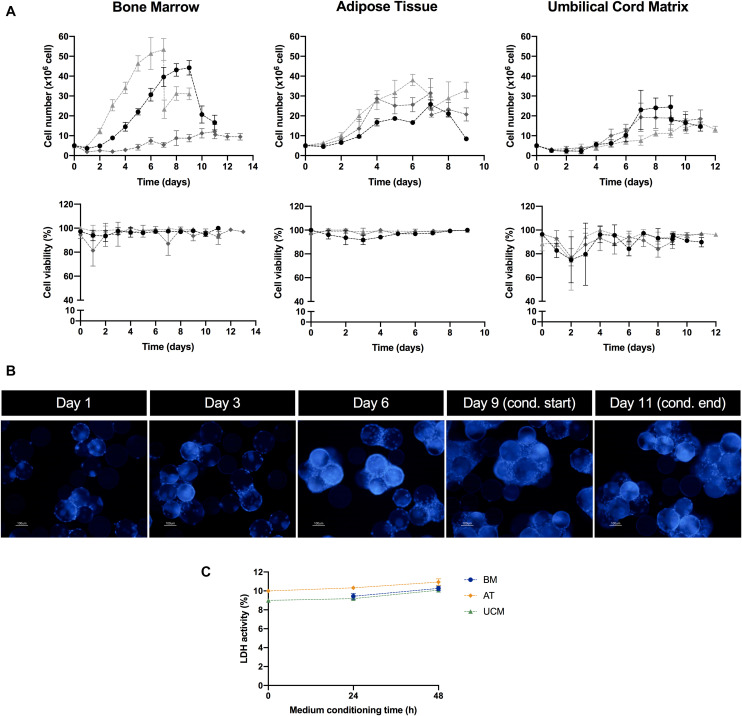
MSC culture in the microcarrier-based bioreactor system. **(A)** Evolution of cell number (upper panel) and cell viability (lower panel) over culture period time, for MSC from three different human tissue sources (bone marrow, adipose tissue, and umbilical cord matrix). MSC from three different donors (i.e., three biological replicates) were used per tissue source, which are represented in three different shades of gray. Two data points are presented for the same day when the medium conditioning stage (i.e., EV production) started. Results are presented as mean ± SD of cell count for each time point. **(B)** Representative images of microcarrier occupation by MSC throughout culture. Cell nuclei were stained with DAPI and images were acquired using a fluorescence microscope. In this case, EV production started on day 9 of culture and finished on day 11. Scale bar = 100 μm. **(C)** LDH activity profile during the medium conditioning (i.e., EV production) stage in the VWBR system. Culture medium samples were taken at 0, 24, and 48 h after medium conditioning started. Results from one experiment for each MSC source (BM, AT, and UCM). Results are presented as mean ± SD (*n* = 3). LDH, lactate dehydrogenase; VWBR, Vertical-Wheel^TM^ bioreactor; BM, bone marrow; AT, adipose tissue; UCM, umbilical cord matrix.

BM, AT and UCM MSC were successfully expanded in the VWBR system (Figure 2A, upper panel). The expansion of BM MSC was the most heterogeneous among donors (*n* = 3), with final post-expansion cell numbers ranging between 12.0 ± 3.6 × 10^6^ and 53.4 ± 5.5 × 10^6^, depending on BM donor. The expansion culture period also ranged from 7 to 11 days in BM MSC cultures. AT and UCM MSC expansion curves were more homogeneous, reaching an average of 29.2 ± 1.7 × 10^6^ and 19.9 ± 2.4 × 10^6^ cells, respectively, at the end of the expansion period. This expansion period was 7 days for AT MSC and 9–10 days for UCM MSC.

Estimated adhesion efficiency of MSC to microcarriers after VWBR inoculation was higher for AT MSC (110 ± 12%), followed by BM MSC (68 ± 17%) and UCM MSC (55 ± 4%) (Table 1). AT MSC adhered and started proliferating in less than 24 h, which resulted in estimated adhesion efficiencies higher than 100%. BM MSC showed the highest average growth rate (0.47 ± 0.05 day^–1^), which was very similar to AT MSC (0.45 ± 0.06 day^–1^), while UCM MSC showed the lowest growth rate (0.35 ± 0.09 day^–1^), as a consequence of the lower initial adhesion efficiency observed.

**TABLE 1:** Parameters from cultures of MSC from three different human sources (BM, AT, and UCM) in bioreactors.

	**Adhesion efficiency**	**Growth rate (day**^–1^)	**Duplication time (day)**
BM	6817%	0.47 ± 0.05	1.49 ± 0.13
AT	11012%	0.45 ± 0.06	1.60 ± 0.19
UCM	554%	0.35 ± 0.09	2.30 ± 0.61

*Average initial cell adhesion efficiency, growth rate and duplication time for each MSC source. Adhesion efficiency was estimated by dividing the total cell number 24 h after inoculation (day 1) by the cell number used in bioreactor inoculation (day 0). Three biological replicates (i.e., MSC from three different human donors) were used for each MSC source (*n* = 3). Results are presented as mean ± SEM.*

In general, BM and AT MSC maintained cell viability close to 100% throughout culture (Figure 2A, lower panel). Cell viability suffered more oscillations in UCM MSC cultures, especially in the first days of culture.

Throughout the culture period, microcarrier colonization by cells increased progressively as MSC expanded (Figure 2B). The increasing microcarrier occupancy was followed by microcarrier aggregation, as MSC expansion reached higher cell numbers. We observed that cell expansion stopped when large microcarrier aggregates were formed, likely due to lack of surface available to attach and proliferate (Figures 2A,B).

In some cultures, a significant decrease in cell number was observed at the start of the medium conditioning stage, immediately after the culture medium was changed from hPL-supplemented medium to supplement-free culture medium. This can be explained, at least partially, by a possible removal of microcarriers during medium change operation, resulting in a loss of cells from the vessel. Additionally, it should be noticed that microcarrier aggregation might affect our estimation of cell numbers at this stage. In the medium conditioning stage, MSC were cultured for 48 h in a supplement-free medium, which could be a stress factor for cell culture. Although a decrease in the cell number was occasionally observed during the 48 h medium conditioning period, this was an exception rather than the rule (Figure 2A). High cell viabilities were maintained (Figure 2A) and there were no visible differences in microcarrier occupancy during this stage (Figure 2B). Still, in order to thoroughly assess if MSC were experiencing induced cell stress, the levels of LDH activity in culture were monitored during the 48 h conditioning period. LDH activity can be used as a readout of cell stress, as this toxic compound is released to cell culture medium upon plasma membrane damage (Racher et al., 1990). LDH activity did not change significantly over this period for any of the MSC sources (Figure 2C). Therefore, there were no indications that MSC were experiencing significant stress in stirred culture due to the absence of hPL in the 48 h conditioning period.

### Characterization of MSC-EVs Reveals Improved Properties Upon Bioreactor Manufacturing

**FIGURE 3:**
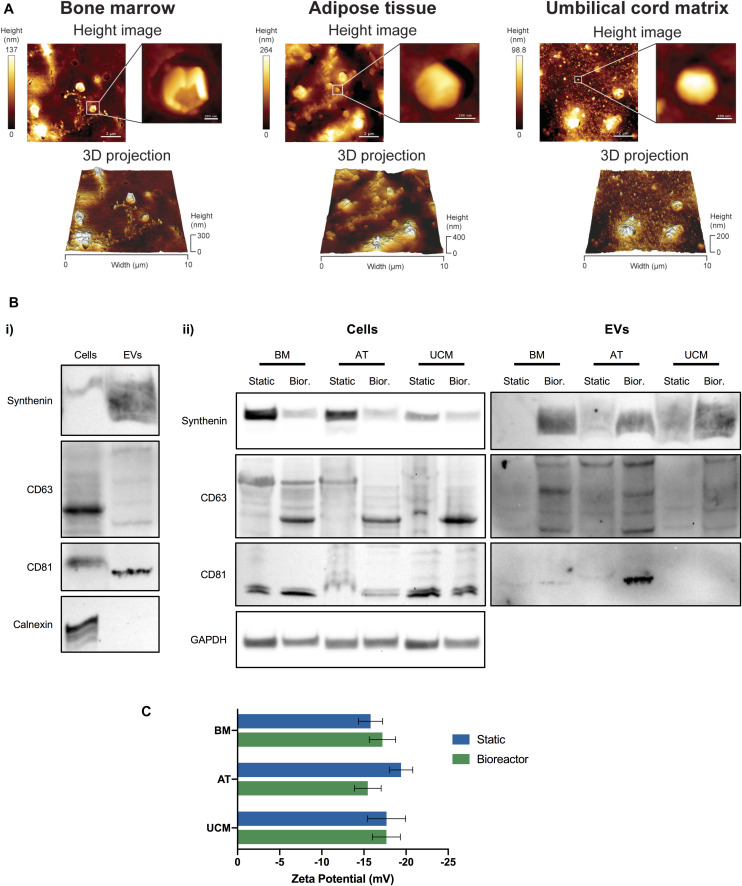
Characterization of MSC-EVs. **(A)** Representative AFM images of MSC-EVs obtained in the VWBR system, using MSC from three different human tissue sources (bone marrow, adipose tissue, and umbilical cord matrix). AFM height images (top) and respective 3D projections (bottom), capturing a total area of 10 × 10 μm. A close-up image focusing on a single EV is presented for each AFM height image. **(B)** Western blots of MSC lysates and MSC-EV samples. (i) Representative Western blot images of synthenin, CD63, CD81 and calnexin detection in MSC-EVs and corresponding WCL (i.e., cells) obtained from VWBR cultures. (ii) Western blot detection of synthenin, CD63 and CD81 in MSC-EV samples and corresponding WCL (i.e., cells), obtained from BM, AT and UCM MSC after EV production in static and VWBR systems. Detection of the housekeeping protein GAPDH in the same WCL preparations. **(C)** Zeta potential measurements of the surface charge of MSC-EVs (mV), obtained in either static or VWBR systems, using MSC from three different human sources (BM, AT, and UCM). Results correspond to one representative experiment for each condition. Results are presented as mean ± SD. AFM, atomic force microscopy; WCL, whole cell lysates; BM, bone marrow; AT, adipose tissue; UCM, umbilical cord matrix; VWBR, Vertical-Wheel^TM^ bioreactor.

EV were successfully isolated from the conditioned medium of MSC cultures. We were able to identify the presence of EVs from static and bioreactor cultures of MSC, from the 3 different sources (i.e., BM, AT and UCM) through AFM (Figure 3A and Supplementary Figure 1). Individual vesicles of different sizes were observed, as well as vesicle aggregates. The formation of aggregates and collapsed vesicles may be caused by sample processing techniques, which involve sample dehydration. Larger vesicles were observed for AT MSC (Figure 3A). These vesicles may have a higher tendency to aggregate or even fuse together due to the higher medium viscosity observed in AT MSC cultures.

The production of EVs was also confirmed by Western blot analysis (Figure 3B and Supplementary Figure 2). The EV protein markers synthenin, CD63 and CD81 were successfully detected in EV samples, while the negative EV protein marker calnexin (a protein from the endoplasmic reticulum) was present in cells, but absent in EV samples, as expected (Figure 3Bi). In general, synthenin and CD63 presence were verified for MSC-EVs obtained from both static and bioreactor systems, using MSC from the 3 different tissue sources (Figure 3Bii). Interestingly, both synthenin and CD63 presence were increased when EVs were obtained from bioreactors. Contrarily to EVs, cells showed higher synthenin expression under static conditions compared to the bioreactor. CD81 was detected in EVs obtained from BM and AT MSC obtained from both static and bioreactor systems, but not from UCM MSC. CD81 was detected in higher quantity in EVs obtained from AT MSC cultured in bioreactors, compared with static conditions.

The surface charge of MSC-EVs was also quantified. MSC-EVs presented a negative surface charge, as determined through zeta potential measurements (Figure 3C). Overall, no significant differences were observed in the zeta potential between samples obtained from static or bioreactor platforms, neither between different MSC tissue sources. The zeta potentials ranged between −15.5 ± 1.6 mV and – 19.4 ± 1.4 mV.

**FIGURE 4:**
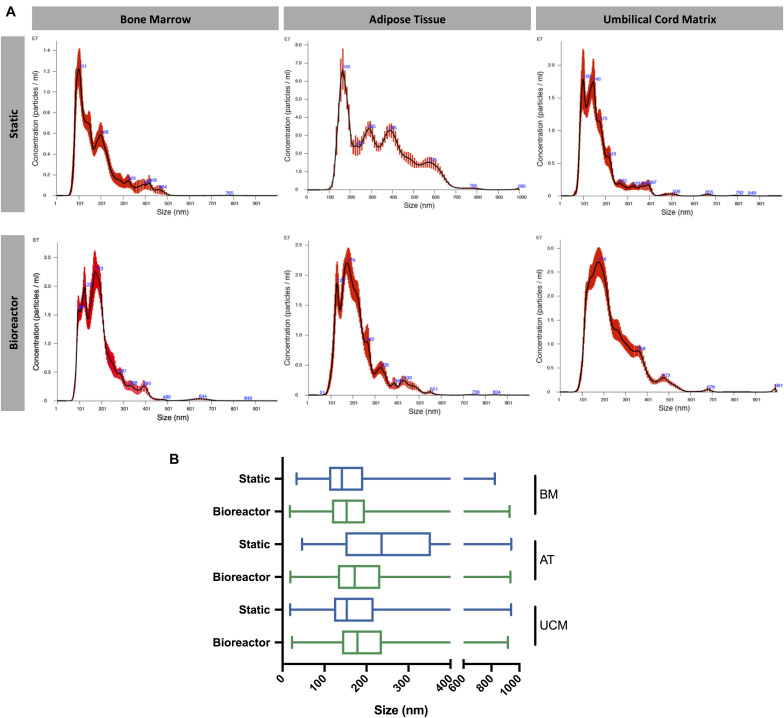
Size distribution of MSC-EVs. **(A)** Representative size distribution curves of EV samples obtained from BM, AT, and UCM MSC, cultured in static or Vertical-Wheel^TM^ bioreactor systems. **(B)** Box plots representing the size distribution profiles of EV samples obtained from BM, AT, and UCM MSC, cultured in static or Vertical-Wheel^TM^ bioreactor systems. The minimum, 1st quartile, median, 3rd quartile and maximum values are represented for each condition. MSC from three different donors were used for each tissue source (i.e., *n* = 3 biological replicates). BM, bone marrow; AT, adipose tissue; UCM, umbilical cord matrix.

The size distribution of MSC-EVs was determined by NTA. In general, MSC-EV samples showed a size distribution profile mostly enriched in small EVs (<200 nm) (Figures 4A,B). Although EVs derived from AT MSC showed a more homogeneous size distribution when obtained from the bioreactor compared to static cultures, no significant difference was observed for other MSC sources. The sizes of EVs produced from AT MSC in the static platform were significantly larger, possibly due to vesicle aggregation or fusion. Therefore, the bioreactor system reveals potential to produce EVs with lower size dispersity, as observed for AT MSC-EVs.

### Bioreactor Culture Improves the Production of MSC-EVs

**FIGURE 5:**
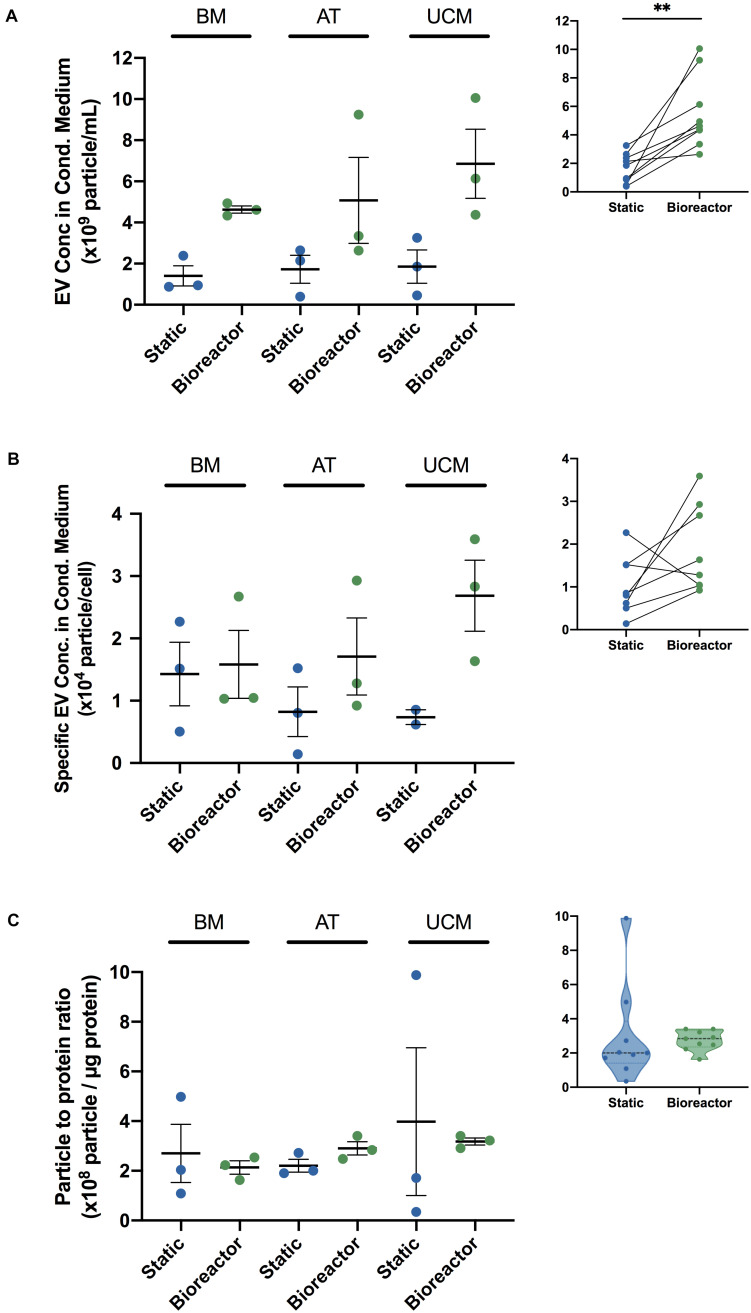
Comparing MSC-EV production in bioreactor and static culture systems, using MSC from different sources. **(A)** EV concentration (particles/mL) in the cell culture conditioned medium from BM, AT, and UCM MSC cultures in static and Vertical-Wheel^TM^ bioreactor systems. MSC from three different donors were used for each tissue source (i.e., *n* = 3 biological replicates). Results are presented as mean ± SEM (*n* = 3). Upper-right panel: Summarized paired analysis comparing EV concentration in static and Vertical-Wheel^TM^ bioreactor systems, for each MSC donor. Paired statistical analysis (paired *t*-test ^∗∗^*P* = 0.0027) (*n* = 9). **(B)** Specific EV concentration (particles/cell) in the cell culture conditioned medium from BM, AT, and UCM MSC cultures in static and Vertical-Wheel^TM^ bioreactor systems. MSC from three different donors were used for each tissue source. In static cultures, each T-175 yielded 1.2 – 6.6 × 10^6^ cells upon 4 – 9 days of expansion, regardless of the cell tissue source. Results are presented as mean ± SEM (*n* = 3; *n* = 2 for UCM-static). Upper-right panel: Summarized paired analysis comparing specific EV concentration in static and Vertical-Wheel^TM^ bioreactor systems, for each MSC donor. **(C)** Particle to protein ratios (PPR) (particle/μg protein) of EV samples obtained from BM, AT and UCM MSC, cultured in static and Vertical-Wheel^TM^ bioreactor systems. MSC from three different donors were used for each tissue source. Results are presented as mean ± SEM (*n* = 3). Upper-right panel: Violin plot of PPR of MSC-EV samples obtained in static and Vertical-Wheel^TM^ bioreactor systems.

MSC-EVs produced in the bioreactor system were quantified by NTA after EV isolation and compared with MSC-EVs obtained from static cultures. When EVs were produced in the bioreactor system, their concentration was significantly increased (Figure 5A), at an overall fold increase of 5.7 ± 0.9 (Table 2). When analyzed individually, we observed a fold increase of 4.0 ± 0.6 for BM MSC, 4.4 ± 1.2 for AT MSC and 8.8 ± 3.8 for UCM MSC, when EVs were produced in the bioreactor system (Table 2). Bioreactor cultured UCM MSC yielded the highest average EV concentration in the conditioned medium (6.9 ± 1.7 × 10^9^ particles/mL) (Figure 5A). The average EV concentration in bioreactor cultures was similar for BM and AT MSC (4.6 ± 0.2 × 10^9^ and 5.1 ± 2.1 × 10^9^ particles/mL, respectively), although the latter presented higher heterogeneity between experiments.

**TABLE 2:** Fold changes in EV concentration and EV productivity in the cell culture conditioned medium from the bioreactor system compared to static conditions.

	**EV concentration fold change (bioreactor/static)**	**EV productivity fold change (bioreactor/static)**
**BM**	4.0 ± 0.6	1.4 ± 0.3
**AT**	4.4 ± 1.2	3.7 ± 1.0
**UCM**	8.8 ± 3.8	3.9 ± 1.4
**Global**	5.7 ± 0.9	3.0 ± 0.5

*Results from each of the 3 MSC sources used (BM, AT, and UCM), as well as global fold change averages from all the sources. Three biological replicates (i.e., MSC from three different human donors) were used for each MSC source (*n* = 3). For each MSC source, results are presented as the average of fold changes for each donor, in order to account for biological diversity. Global fold changes are presented as the average of fold changes from each MSC source. Results are presented as mean ± SEM.*

In order to evaluate if the conditions in the bioreactor might modulate the intrinsic capacity of cultured MSC for the production of EVs compared to static conditions, we estimated the EV productivity (i.e., specific EV concentration, per cell) by dividing the concentration of EVs (from NTA) by the cell concentration at the beginning of the conditioning period. When EVs were produced in the bioreactor system, EV productivity increased compared with static culture (Figure 5B) at an overall fold increase of 3.0 ± 0.5 (Table 2). Although this difference was not statistically significant (which is likely due to the heterogeneities between the different tissue sources and donors used), the bioreactor system allowed an improved productivity of MSC-EVs for most of the MSC donors used (i.e., in six out of eight MSC donors).

EV productivity increased in the bioreactor by a fold increase of 1.4 ± 0.3 for BM MSC, 3.7 ± 1.0 for AT MSC and 3.9 ± 1.4 for UCM MSC (Table 2), compared with static conditions. Bioreactor cultured UCM MSC yielded the highest average EV productivity (2.7 ± 0.6 × 10^4^ particles/cell) (Figure 5B). The average EV productivity in bioreactor cultures was similar for BM and AT MSC (1.6 ± 0.5 × 10^4^ and 1.7 ± 0.6 × 10^4^ particles/cell, respectively).

A particle to protein ratio (PPR) was also determined by dividing the EV concentration (determined by NTA) by the total protein concentration in the same sample (determined through BCA protein assay). The PPR can be used to assess the purity of an EV sample, as the higher is this ratio, the lower is the amount of co-isolated protein contaminants, thus the higher is the sample purity (Webber and Clayton, 2013). EV samples from BM and UCM MSC cultures presented a more homogeneous PPR in the bioreactor system than in static conditions (Figure 5C). EV samples from AT MSC cultures presented a homogeneous PPR for both culture platforms, but the average PPR was slightly higher in the bioreactor. Overall, the PPR was relatively constant in the bioreactor system, ranging between 1.63 × 10^8^ and 3.40 × 10^8^ particles/μg protein (Figure 5C). PPR was much more heterogeneous in static conditions (i.e., T-flasks), ranging between 3.47 × 10^7^ and 9.88 × 10^8^ particles/μg protein. Additionally, the median PPR was higher for the EVs produced in the bioreactor system.

## Discussion

Mesenchymal stromal cells hold great promise for the development of cell-based therapies for a variety of disorders. MSC-derived products such as MSC-EVs offer the opportunity to develop new therapeutic products benefiting from MSC regenerative properties in cell-free formulations. These cell-free therapies are expected to present significant advantages, obviating the complexity and safety issues in utilizing cells themselves as therapeutic systems in a clinical context (Batrakova and Kim, 2015; Conlan et al., 2017).

MSC-EVs can be used as intrinsically therapeutic products, by mediating some of the effects conveyed by MSC. MSC-EVs present therapeutic properties for neurological, cardiovascular, immunological, kidney and liver diseases, among others (Phinney and Pittenger, 2017; Keshtkar et al., 2018; Elahi et al., 2020). MSC-EVs have been described to reduce myocardial ischemia/reperfusion injury in mice (Lai et al., 2010) and also allowed improved recovery from acute kidney injury (Bruno et al., 2012) and from stroke (Doeppner et al., 2015). Indeed, there are multiple studies describing their pro-angiogenic (Bian et al., 2014; Vrijsen et al., 2016) and wound healing capacity (Zhang et al., 2015; Fang et al., 2016).

Alternatively, EVs can be engineered toward the development of novel drug delivery systems (DDS). Drug loaded EVs can be used to transport and deliver therapeutic cargo to target diseased cells and tissues (Batrakova and Kim, 2015; Vader et al., 2016). These natural DDS could be an appealing alternative to the more established synthetic DDS, by avoiding toxicity and rapid clearance from the organism, as well as a better membrane matching capacity (Batrakova and Kim, 2015). Dendritic cell-derived EVs were able to deliver siRNA to the brain in mice, demonstrating their potential use as targeted therapy for neurological diseases (Alvarez-Erviti et al., 2011). Macrophage-derived EVs loaded with catalase provided increased neuroprotective effects in *in vitro* and *in vivo* models of Parkinson’s disease, compared to free catalase (Haney et al., 2015). Recently, multiple studies have successfully developed EVs as DDS for cancer therapy (Pascucci et al., 2014; Tian et al., 2014; Kim et al., 2016, 2018; Kooijmans et al., 2016, 2018; Jia et al., 2018; Li et al., 2018). Intravenously injected EVs from dendritic cells delivered doxorubicin specifically to tumor tissues in mice, leading to the inhibition of tumor growth with lower toxcicity (Tian et al., 2014). MSC incubated with a high Paclitaxel concentration secreted EVs loaded with this drug, successfully inhibiting tumor growth *in vitro* (Pascucci et al., 2014). Additionally, EVs can be further engineered to improve specificity and retention on target cells and tissues (Jia et al., 2018; Kooijmans et al., 2016, 2018).

Despite the promising potential of EVs for therapeutic applications, large EV doses are expected to be required to achieve therapeutic effects in clinical settings. This requires the development of robust manufacturing processes that could increase the consistency and scalability of EV production, which are currently lacking.

The present work aimed to establish a scalable culture platform for the manufacturing of MSC-EVs in S/XF culture conditions. This was achieved by building on previous work from our group where a S/XF microcarrier-based culture system was implemented in single-use bioreactors (VWBR), employing a hPL culture supplement (UltraGRO^TM^-PURE) for MSC expansion (de Sousa Pinto et al., 2019). In the present study, EVs were produced by MSC isolated from 3 different human tissue sources (BM, AT and UCM) in a process that comprises a cell expansion stage and a culture medium conditioning stage.

S/XF culture conditions were implemented by exclusively applying products without any animal components, namely hPL as a culture supplement used in the cell expansion stage, instead of the more commonly used FBS, as well as animal product-free plastic microcarriers and TrypLE as a cell detaching solution. Multiple studies have revealed hPL-supplemented media to be efficient for the isolation and expansion of MSC from various origins (Doucet et al., 2005; Kinzebach et al., 2013; Reinisch et al., 2015), cultured both in static and dynamic systems (de Soure et al., 2017; de Sousa Pinto et al., 2019), as well as for the expansion of other cell types (Naveau et al., 2010; Mazzocca et al., 2012; Hofbauer et al., 2014; Hildner et al., 2015). However, the fact that hPL products originate from human donors presents some constraints, such as the risk of transmission of human diseases by viruses, ill-definition and the possibility of triggering immune responses (Hemeda et al., 2014). The ideal option for production of clinical-grade cell based therapies would be a chemically defined, animal component-free medium (including human). However, there are very few of these options available, namely for MSC culture. Therefore, presently, hPL seems to be the most promising and cost-effective alternative to FBS supplementation in cell culture medium for now, being more readily translatable to a clinical setting, especially considering that gamma irradiated hPL products allowing significant viral reduction have already been developed (Huang et al., 2019).

Culture medium supplements such as FBS and hPL have a large amount of protein and vesicle contents, presenting an additional challenge for their use in EV manufacturing. These components are prone to be co-isolated with the EV fraction, thus contaminating the end product (Witwer et al., 2019). For this reason, we removed hPL at the end of the MSC expansion period and hPL-free medium was used for the medium conditioning period. MSC were cultured for 48 h in this supplement-free medium, which could be a stress factor for cell culture. However, we did not observe any significant reduction in cell number, cell viability or microcarrier occupancy during this stage. Furthermore, LDH activity did not change significantly over this period for any of the MSC sources. Therefore, there were no indications that MSC were experiencing significant stress in culture, due to the absence of hPL in the 48 h conditioning period. Still, MSC might potentially undergo some alterations over this period. Minimal identity criteria commonly used to define multipotent MSC could suffer modifications, namely their *in vitro* multilineage differentiation capacity or their immunophenotype (i.e., expressing CD73, CD90, and CD105, lacking the expression of hematopoietic and endothelial markers CD11b, CD14, CD19, CD34, CD45, CD79a and HLA-DR) (Viswanathan et al., 2019). Of notice, MSC expanded in the VWBR system maintain the typical MSC immunophenotype, as previously reported by our group (de Sousa Pinto et al., 2019). Further work could be performed by comparing the MSC features before and after the culture medium conditioning period.

Bioreactor systems such as VWBR present several advantages for the manufacturing of cell-based therapies. Cell culture on microcarriers in suspension inside a bioreactor allows an increase of available surface area per volume ratio, enabling higher cell concentrations in culture. Bioreactors also allow the implementation of culture monitoring and control systems, providing an additional advantage to optimize culture conditions, by adjusting feeding regimes and physicochemical parameters (e.g., O_2_ concentration and pH) according to real-time culture measurements.

In this work, we established a bioreactor process in 100 mL VWBR vessels. This process can be scaled-up to VWBR with a working volume of 3 L or higher (up to 500 L), which include an integrated control system, allowing for a controlled manufacturing process. To the best of our knowledge, this study is the first to establish a S/XF microcarrier-based culture system in bioreactors for the manufacturing of MSC-EVs, using MSC from 3 different human tissue sources (BM, AT and UCM). It is also the first to implement the VWBR configuration for EV production. Cell expansion in this bioreactor culture system allowed an increase in EV concentration in the conditioned medium when compared to traditional static systems (5.7 ± 0.9 global fold increase), partly due to higher cell concentrations obtained in VWBR. However, in addition to that, the EV productivity (i.e., specific EV concentration) also increased in bioreactors (3.0 ± 0.5 global fold increase), meaning that each cell secreted more EVs when MSC were cultured in the VWBR, compared to static conditions. Although this difference was not found to be statistically significant, this was likely due to the heterogeneities between different tissue sources and donors. For example, if we had not considered the results from one of the BM MSC donors (for which EV productivity decreased in the bioreactor, contradicting the observed general tendency of our study), this difference would be statistically significant. This reinforces the relevance of testing MSC from multiple tissue donors in order to account for intrinsic biological variability. Of notice, this study was performed using MSC from 3 different donors for each tissue source, comprising a total 9 random human donors. Still, further work may be performed with additional donors in order to more thoroughly account for donor variability and its impact. Altogether, the higher EV concentrations achieved in VWBR were due to higher cell densities, as well as to higher EV productivities by MSC.

Overall, in the conditions of our study, UCM MSC allowed the highest EV concentration and EV productivity in the bioreactor system. They also showed the highest fold increase in both parameters when compared to static systems. Therefore, UCM seems to be the MSC source that benefits the most from cultivation in the VWBR system, being the most promising of the three tissue sources studied for scalable MSC-EV production. This is in line with previous work where UCM MSC have been described to allow higher EV productivity than BM and AT MSC in static culture (Haraszti et al., 2018).

Nonetheless, the real applicability of these MSC-EVs depends on their biological function. Given their different tissue origins, we can expect that EVs obtained from cells derived from each MSC source will have different functional characteristics. Indeed, different intrinsic therapeutic features have been described for MSC derived from different tissues (Ribeiro et al., 2013). In order to develop therapeutic products, based on the MSC-EVs manufactured in this work, additional functional studies will be required. These could include, for example, (i) scratch assays or tube formation assays using endothelial cells to determine the ability of MSC-EVs to promote angiogenesis in the context of vascular repair (Vrijsen et al., 2016) or (ii) cell uptake assays to determine EV uptake by target cancer cells, to assess their potential as drug delivery vehicles for cancer therapy (Kooijmans et al., 2018).

The increase observed in EV productivity in VWBR can be explained by multiple reasons. EV secretion by MSC may have been stimulated by fluid flow, promoted by the VWBR mixing system. Fluid flow has already been described to stimulate EV secretion in osteocytes through a Ca^2+^-mediated response (Morrell et al., 2018). Additionally, when MSC were cultured in the bioreactor system, cells attached to the surface of plastic microcarriers and proliferated. Later in culture, microcarrier aggregates were formed and, consequently, MSC formed aggregates as well, as previously observed (Frauenschuh et al., 2007; Eibes et al., 2010). MSC culture in spheroids has been described to lead to higher secretion of paracrine factors (Bhang et al., 2011; Costa et al., 2017), as well as to an increased secretion of microvesicles (Cha et al., 2018). Hence, aggregate formation could be leading to an increased EV secretion in the VWBR system. Finally, MSC cultured in the VWBR system are likely to be exposed to lower oxygen concentrations than in static platforms. The VWBR agitation system allows mixing of the cell culture medium, achieving a homogeneous oxygen concentration. However, there is no aeration system in the 100 mL VWBR, so oxygen exchange occurs only at the surface gas-liquid interface. Considering the differences between the geometries of the VWBR vessel and the T-flask, oxygen concentration would be expectedly lower in the VWBR system than in static. This could potentially be a contributing factor for the observed increase in EV secretion when cells were expanded in the bioreactor system. Previous studies have demonstrated an increase in EV secretion when different cell types (including MSC) were cultured under hypoxic conditions (ranging from 0.1 to 3% O_2_, compared to controls) (King et al., 2012; Salomon et al., 2013; Panigrahi et al., 2018). Although all of these factors might lead to an increased EV productivity in the VWBR, additional studies would be needed to determine their actual contributions.

Zeta potential measurements revealed that the surface charge of obtained MSC-EVs were generally similar, regardless the production platform and MSC source used, ranging between −15.5 ± 1.6 mV and −19.4 ± 1.4 mV. These surface charges are moderately negative, as it was expected considering that EVs are cell-derived nanoparticles, therefore containing negatively charged phospholipids. The values of zeta potential obtained herein were in line with other studies reporting zeta potential measurements for EVs derived from cell culture (Akagi et al., 2014; Hood et al., 2014; Kesimer and Gupta, 2015; Rupert et al., 2017).

Further EV characterization revealed that bioreactors improved not only EV quantity but also their purity, as assessed by Western blot and PPR. Western blot analysis revealed that synthenin, CD63 and CD81 (key proteins involved in EV biogenesis and commonly used as protein markers) were in general more abundant in EVs obtained from bioreactors than from their static counterparts (Figure 3Bii). Therefore, EVs from bioreactors seem to have a higher purity than EVs obtained from static system, since a higher amount of synthenin, CD63 and CD81 were detected for the same amount of total protein. This observation corroborates the increased EV concentration in VWBR identified by NTA. The fact that bioreactor EV samples showed increased levels of EV protein markers validates the hypothesis that the increased concentration of particles detected by NTA corresponds to an increased concentration of EVs and not of protein aggregates.

EV purity was also assessed by estimating the PPR for each EV sample (Webber and Clayton, 2013). PPR was more homogeneous and reproducible in EV samples obtained from bioreactors compared to those produced under static conditions and the median PPR was higher in the bioreactor system (Figure 5C). A more homogeneous environment in VWBR offers a more reproducible process for different sources and donors. Constant agitation provides the cells with a more homogeneous access to nutrients, thus allowing a more robust MSC-EV manufacturing process. Therefore, the bioreactor platform established in this work is expected to allow the robust production of MSC-EVs at higher purities, compared to static systems.

In our previous work focused on the establishment of a S/XF microcarrier-based culture system in single-use bioreactors (VWBR) (de Sousa Pinto et al., 2019), an economic evaluation revealed that the application of this culture system allowed a cost reduction for MSC manufacturing when compared to static cell culture using T-flasks. Therefore, it can be expected that the application of this bioreactor system will also allow a cost reduction for the production of MSC-EVs, compared to static platforms.

A few manufacturing processes for the production of EVs have been previously studied. The Integra CELLine culture system is a static platform that has been used to optimize EV production (Mitchell et al., 2008). This is a two-compartment culture flask with a semi-permeable membrane separating a cell-containing compartment from a larger medium compartment. When mesothelioma and NK cells were cultured in this system, a 12-fold and a 8-fold increase in EV (protein) concentration was observed, respectively, compared to traditional T-flasks (Mitchell et al., 2008). This system also allowed a 13- to 16-fold increase in EV (protein) concentration from bladder carcinoma cells (Jeppesen et al., 2014). The CELLine system allows culture medium change while EVs are retained in the cell compartment, enabling higher EV concentrations. However, this static system has limited scalability, thus not being the most suitable option for large-scale EV production.

Watson and colleagues developed a hollow-fiber bioreactor platform for the production of HEK-derived EVs (Watson et al., 2016). The authors reported a 10-fold increase in EV concentration compared with static culture, which was sustained by an increased purity (both increased PPR and protein marker expression). However, EV size distribution profiles were more dispersed in the bioreactors, which is the opposite from what we observed in our study with the VWBR system. Mendt and colleagues manufactured BM MSC-derived EVs in a closed system, hollow-fiber bioreactor, named Quantum (Mendt et al., 2018). They were able to achieve 1.04 × 10^10^ particles/mL on average, which was higher, but comparable with the EV concentrations we obtained in the VWBR system (5.5 ± 0.8 × 10^9^ particles/mL) herein.

Hollow-fiber bioreactors (i.e., without mechanical agitation) provide surface immobilization of cells on the fibrous material and represent a suitable configuration to obtain an increased EV concentration in culture, since culture medium can be recirculated while EVs are retained by the hollow-fiber membranes. However, stirred bioreactors as the VWBR may allow a better fine-tuning of EV production by manipulating process parameters. For example, agitation may play an important role in EV secretion, since fluid flow seems to have impact on this process. Further studies may be developed in the VWBR, testing the impact of agitation on EV production. Other process parameters, such as oxygen concentration, temperature and pH, are also likely to play a role in EV secretion by cultured MSC and are more easily controlled in a VWBR, especially when integrated with a control system. Further studies addressing the impact of these parameters on EV production using the VWBR system would be relevant to fine-tune and optimize MSC-EV production.

## Conclusion

In this study, we have successfully developed a scalable S/XF microcarrier-based bioreactor culture system for the robust production of MSC-EVs, using MSC from 3 different human tissue sources (BM, AT, and UCM). This system allowed the production of MSC-EVs at higher concentration and productivity when compared to traditional static culture systems. It also allowed to obtain a more robust MSC-EV manufacturing process, regarding their purity. Further developments of this system will need to take into consideration a proper balance between EV production and function. Additional studies will be required to characterize the therapeutic potential of these MSC-EVs. The MSC-EVs obtained through this scalable platform are promising for the development of multiple therapeutic products and DDS, targeting a variety of diseases.

## Data Availability Statement

The original contributions presented in the study are included in the article/Supplementary Material, further inquiries can be directed to the corresponding authors.

## Author Contributions

MAF, NB, AF-P, DG, JC, and CS designed the research study. MAF performed the MSC-EV production and isolation. MAF and NB performed the characterization and data analysis. AC assisted on MSC-EV production. AF-P supported the establishment and management of the MSC bank. FO, MC, and DG were responsible for zeta potential and AFM characterization. JF assisted on NTA. CR, SJ, and BL supported the development of the Vertical-Wheel^TM^ bioreactor system. R-JT and WM supported the use of the human platelet lysate culture supplement (UltraGRO^TM^-PURE) for cell isolation and expansion. MAF, NB, and CS wrote the manuscript. All the authors critically revised and approved the final manuscript.

## Conflict of Interest

SJ is employee of PBS Biotech, Inc. BL is CEO and co-founder of PBS Biotech, Inc. These collaborating authors participated in the development of the bioreactor systems used in the manuscript. R-JT and WM are employees of AventaCell Biomedical Corp. These collaborating authors participated in the development of the culture medium supplement used in the manuscript. This does not alter the authors adherence to all the policies of the journal on sharing data and materials. The remaining authors declare that the research was conducted in the absence of any commercial or financial relationships that could be construed as a potential conflict of interest.
