# Supplementary material for: Scalable Production of Human Mesenchymal Stromal Cell-Derived Extracellular Vesicles Under Serum-/Xeno-Free Conditions in a Microcarrier-Based Bioreactor Culture System
Source: Front Cell Dev Biol. 2020 Nov 3;8:553444. doi: 10.3389/fcell.2020.553444 (PMC7669752; doi:10.3389/fcell.2020.553444)
Supplement: Supplementary file 1 [file Table_1.DOCX]

Supplementary Material

**Supplementary Figure 1**


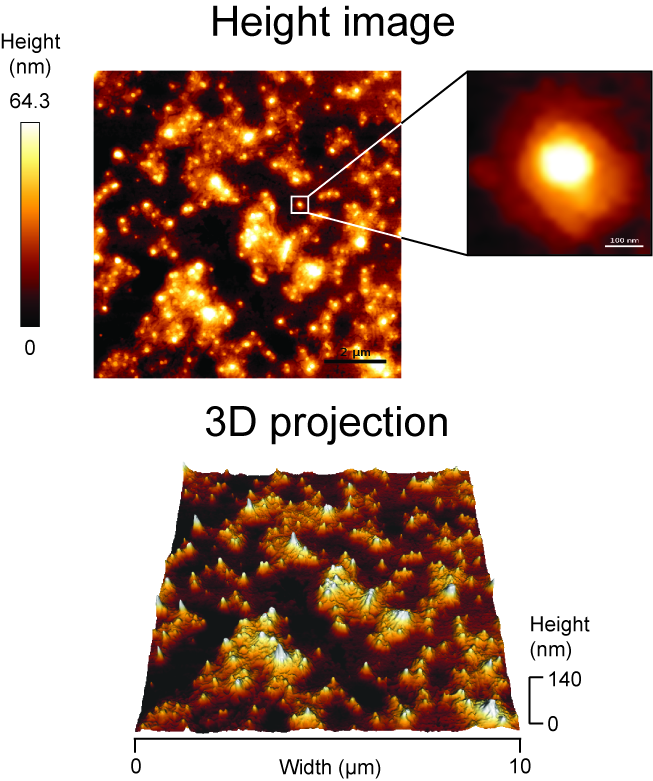


**Supplementary Figure 1 -** Representative AFM image of MSC-EVs obtained in static conditions (in this particular case EVs were obtained from AT MSC cultures). AFM height image (top) and respective 3D projection (bottom), capturing a total area of 10 x 10 μm. A close-up image focusing on a single EV is presented. AFM - atomic force microscopy.

**Supplementary Figure 2**


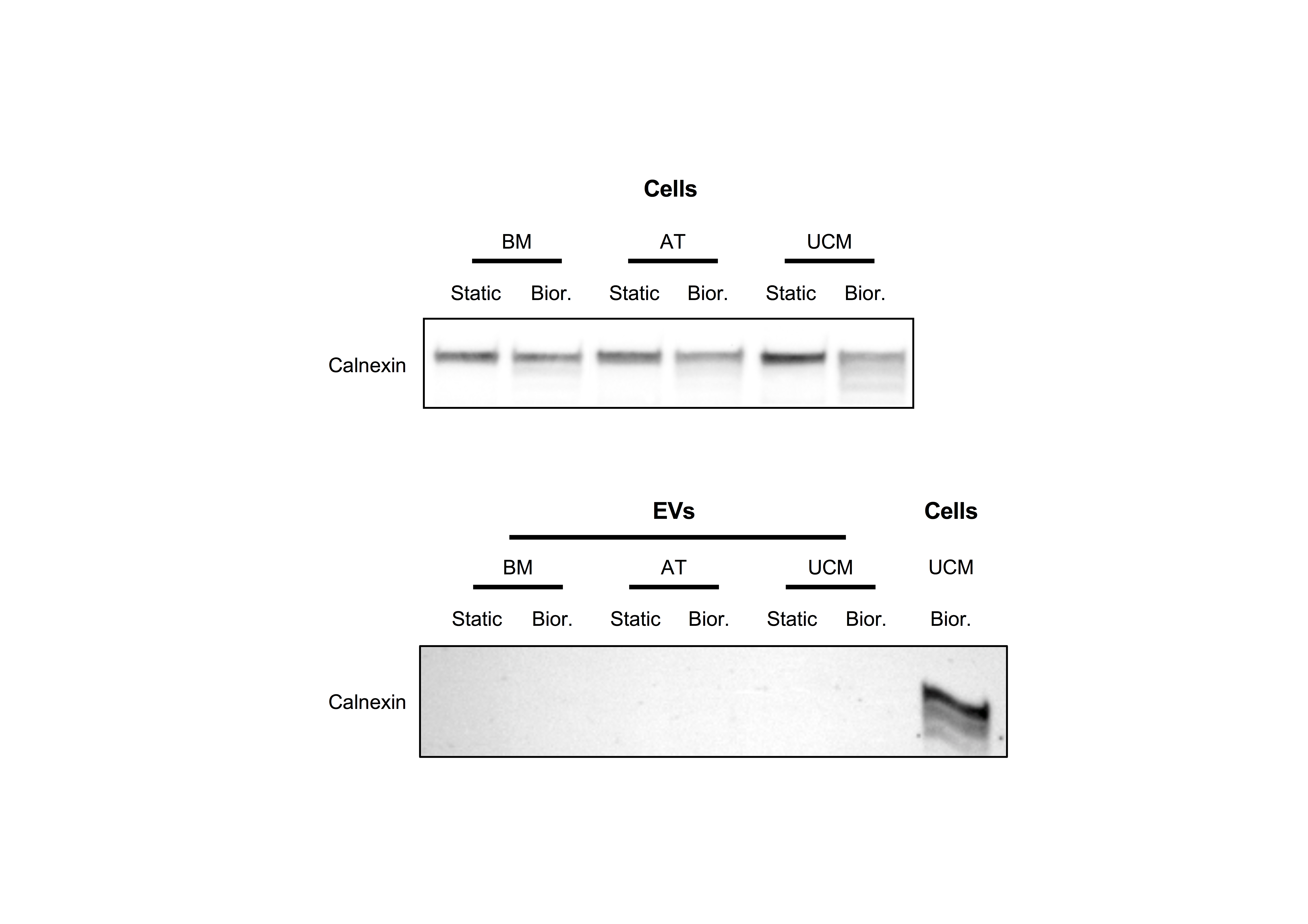


**Supplementary Figure 2 -** Western blot detection of calnexin in MSC lysates (*i.e.* cells) and MSC-EV samples obtained from BM, AT and UCM MSC after EV production in static and Vertical-Wheel^TM^ bioreactor systems. BM - bone marrow. AT - adipose tissue. UCM - umbilical cord matrix.
